# Breathing Pattern Disorders Distinguished from Healthy Breathing Patterns Using Optoelectronic Plethysmography

**DOI:** 10.1155/2022/2816781

**Published:** 2022-12-03

**Authors:** Carol M. E. Smyth, Samantha L. Winter, John W. Dickinson

**Affiliations:** ^1^School of Sport and Exercise Sciences, University of Kent, Chipperfield Building, Canterbury Kent CT2 7NZ, UK; ^2^School of Sport, Exercise and Health Sciences, Loughborough University, National Centre for Sport and Exercise Medicine, Loughborough LE11 3TT, UK

## Abstract

There is no gold standard diagnostic method for breathing pattern disorders (BPD) which is commonly diagnosed through the exclusion of other pathologies. Optoelectronic plethysmography (OEP) is a 3D motion capture technique that provides a comprehensive noninvasive assessment of chest wall during rest and exercise. The purpose of this study was to determine if OEP can distinguish between active individuals classified with and without BPD at rest and during exercise. Forty-seven individuals with a healthy breathing pattern (HBP) and twenty-six individuals with a BPD performed a submaximal exercise challenge. OEP measured the movement of the chest wall through the calculation of timing, percentage contribution, and phase angle breathing pattern variables. A mixed model repeated measures ANOVA analysed the OEP variables between the groups classified as HBP and BPD at rest, during exercise, and after recovery. At rest, regional contribution variables including ribcage percentage contribution (HBP: 71% and BPD: 69%), abdominal ribcage contribution (HBP: 13% and BPD: 11%), abdomen percentage contribution (HBP: 29% and BPD: 31%), and ribcage and abdomen volume index (HPB: 2.5 and BPD: 2.2) were significantly (*p* < 0.05) different between groups. During exercise, BPD displayed significantly (*p* < 0.05) more asynchrony between various thoracic compartments including the ribcage and abdomen phase angle (HBP: −1.9 and BPD: −2.7), pulmonary ribcage and abdomen phase angle (HBP: −0.5 and BPD, 0.5), abdominal ribcage and shoulders phase angle (HBP: −0.3 and BPD: 0.6), and pulmonary ribcage and shoulders phase angle (HBP: 0.2 and BPD: 0.6). Additionally, the novel variables inhale deviation (HBP: 8.8% and BPD: 19.7%) and exhale deviation (HBP: −10.9% and BPD: −17.6%) were also significantly (*p* < 0.05) different between the groups during high intensity exercise. Regional contribution and phase angles measured via OEP can distinguish BPD from HBP at rest and during exercise. Characteristics of BPD include asynchronous and thoracic dominant breathing patterns that could form part of future objective criteria for the diagnosis of BPD.

## 1. Introduction

Many respiratory symptoms, such as dyspnoea, chest tightness, and wheezing, are not specific to a single respiratory disease/disorder. These symptoms are also often related to breathing pattern disorder (BPD) and exercise-induced bronchoconstriction (EIB) [1, 2]. Reports suggest that approximately 20% of athletes who report significant exercise respiratory symptoms receive an inappropriate diagnosis of asthma or EIB [3]. A differential diagnosis for these individuals may be BPD.

BPD is the result of inappropriate chest wall movements, which can present with or without another respiratory disease/disorder. Characteristics of these movements have been identified including apical/thoracic dominant breathing [4, 5], thoracoabdominal asynchrony [6], and/or irregular breathing rhythm, which have been used to diagnose BPD [7]. However, there is no formal definition or gold standard diagnostic method or criteria for BPD [6], and it is therefore difficult to establish an accurate prevalence. However, Thomas and colleagues [8] reported that dysfunctional breathing has an occurrence of ∼2% and ∼14% in males and females, respectively, in the general population. It has recently been reported that 20% of elite athletes have symptoms suggestive of BPD [9]. In individuals with asthma, 30–65% also display symptoms associated with BPD [8, 10].

Currently, BPD diagnosis relies on the exclusion of other pathologies using various breathing function measures and may be treated using nonpharmacological strategies [11]. Clinicians utilise a number of resources to diagnose BPD including questionnaires (e.g., breathing pattern assessment tool (BPAT)), and evaluation of breathing pattern techniques (e.g., manual assessment of respiratory motion (MARM)). These assessments aim to identify one or more BPD characteristic or symptom. Despite their extensive use, meaningful clinical differences have not been established for these assessments [12]. Due to the lack of standard diagnostic measures, individuals with BPD often have a delayed diagnosis or a misdiagnosis, with the average length of time until a correct diagnosis being between 2–7 years [13]. Diagnosis is complicated by similarities between other diseases/disorders, the co-existing of BPD with other disease/disorders, and symptoms of BPD may not be present at all times; for example, the symptoms may be exercise- or stress-induced [6]. Assessments such as the MARM can only be utilised statically, making it unsuitable for assessing BPD in athletes during exercise. It is important to diagnose BPD as soon as possible to avoid the incorrect use of medication or unnecessary treatments. When BPD is treated correctly, individuals show improvement in quality of life and reduced breathing difficulties during daily life and exercise [14, 15]. This highlights the need for diagnostic measures to be sensitive and specific enough to distinguish between respiratory diseases/disorders to avoid misdiagnosis and the incorrect/unnecessary use of medication or treatments.

Optoelectronic plethysmography (OEP) is a noninvasive method of objectively assessing breathing pattern. OEP allows for the objective measure of breathing pattern characteristics through the measurement of regional contribution and phase angle variables which allow for apical and asynchronous breathing patterns to be quantified. Unlike other similar techniques such as respiratory inductive plethysmography (RIP) and structured light plethysmography (SLP), OEP can be utilised during movement and exercise, making it suitable for measuring the breathing function of athletes during exercise. Due to the symptom similarities between BPD and other diseases, a focus on the breathing pattern characteristics may be more useful for diagnosis. As BPD can be exercise-induced, it would be expected that symptoms are likely to onset at higher exercise intensities. Individuals with BPD will present with characteristics currently used for diagnosis (e.g., BPAT and RIP) such as apical or asynchronous movement of the chest during exercise [7] but potentially not at rest when compared to individuals with a healthy breathing pattern.

Therefore, the purpose of this study was (1) to establish breathing pattern characteristic for BPD using OEP at rest and during exercise and (2) to investigate if OEP-derived breathing variables, specifically regional contribution and phase angle variables, differ between individuals with a suspected BPD from those without any known breathing disorders.

## 2. Materials and Methods

### 2.1. Participants

Eighty-five active participants (Table 1) gave informed written consent to participate in this study, which was approved by the University of Kent's Research Ethics Advisory Group (Prop 21_2018_19). Participants were active (at least 150 minutes of moderate-intensity, or at least 75 minutes of vigorous-intensity aerobic physical activity, or a combination, each week) and were recreational or club athletes. Participants completed a questionnaire regarding respiratory symptoms during or postexercise and in various environmental conditions. The questionnaire identified symptoms such as coughing, wheezing, chest tightness, dyspnoea, excess mucus during exercise, whether environmental conditions exacerbated symptoms and asked about any prior respiratory pathology diagnosis.

**Figure 1:**
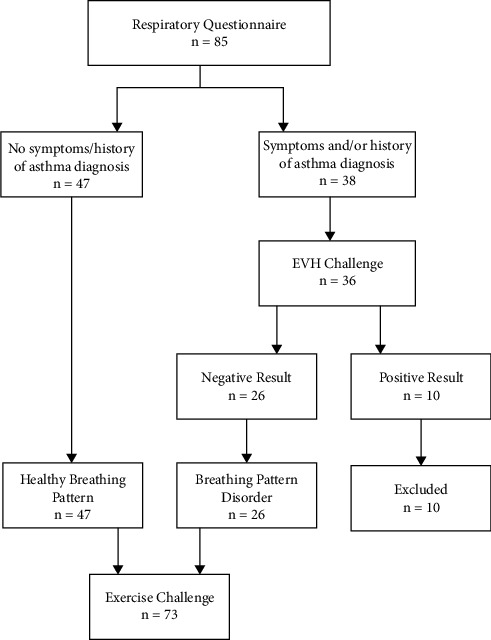
Summary of the study protocol.

Individuals who reported no respiratory symptoms and had no history of any respiratory disease were classified into the healthy breathing pattern group. Individuals who reported respiratory symptoms and/or had a previous/current diagnosis of asthma/EIB performed an eucapnic voluntary hyperpnoea (EVH) challenge [16]. Individuals who demonstrated a negative EVH result (fall in forced expiratory volume in one second of <10% post-EVH) but reported exercise respiratory symptoms and were otherwise healthy with no previous cardio-respiratory diagnosis, were classified into the BPD group. The healthy breathing pattern (HBP) and BPD group performed a submaximal exercise challenge (Figure 1).

### 2.2. Equipment

The OEP system consisted of 11 cameras (Oqus 3, Qualisys AB, Goteborg, Sweden) sampling at 100 Hz and were positioned around a cycle ergometer (Lode-Corival, Netherlands). Ninety reflective markers were placed on the participants' torso in a grid-like pattern [17, 18]. This marker set allows for the division of the torso into the pulmonary ribcage (RCp), the abdominal ribcage (RCa), and the abdomen (AB).

### 2.3. Protocol

Participants began with a period of tidal breathing, followed by a submaximal cycle challenge. The exercise challenge began at 50 W which was increased by 30 W every minute. The exercise intensities used in this study were defined using a rating of perceived exertion (RPE) scale with low, moderate, and high intensity being defined as an RPE values of 11, 13/14, and 17/18, respectively [19]. Once a participant reached each intensity, OEP data was recorded for approximately 30–60 seconds. OEP data was also recorded pre and postexercise challenge to obtain resting and recovery data.

### 2.4. Data Analysis

OEP data was processed using Qualisys Track Manager (v2019.2 Build 4610). Custom-built MATLAB (version R2019a) scripts were used to calculate the OEP breathing variables. The following time-derived variables were calculated: respiratory rate (RR), tidal volume (Vt), minute ventilation $\left({\dot{V}}_{E}\right)$, inspiratory time (tI), expiratory time (tE), and total breath time (tTot).

Regional contribution variables calculated consisted of various compartment contributions to the total volume including the ribcage (RcCT), pulmonary ribcage (RCpCT), abdominal ribcage (RCaCT), abdomen (AbCT), combined RCa and abdomen contribution (RCaAbCT), and the ribcage and abdomen volume index (RcAbIndex).

Phase angle is a measure of the temporal movement of one torso compartment in relation to another during each breath and can be visually represented using Konno-Mead loops. This study calculated the phase angles between the ribcage and the abdomen (RcAbPhase), between the pulmonary ribcage and the combined abdominal ribcage and the abdomen (RCpAbPhase), and between the pulmonary and abdominal ribcage (RCpRCaPhase). Finally, the phase angle between the shoulders and various compartments were calculated including the abdominal ribcage (RCaSPhase), the bottom of the pulmonary ribcage (RCpSPhase), and the abdomen (AbSPhase). Inhale and exhale percentage deviation variables measure the deviation of the phase angle trace from the “perfect” straight 45 degree line within the Konno-Mead loop during the inhale and exhale respectively.

### 2.5. Statistical Analysis

95% upper and lower confidence intervals were calculated for each OEP variable across each condition, i.e., rest, low, moderate, high intensity exercise, and recovery postexercise. A mixed model repeated measures ANOVA was used to analyse differences between exercise condition (rest, recovery, and low, moderate, high intensity) in the breathing variables between the HBP and BPD groups.

## 3. Results

Participants classified into the BPD group reported symptoms including coughing, wheezing breathing in and/or out, chest tightness breathing in and/or out, dyspnoea, and excess mucus production during or postexercise.

Tables 2–4 display the mean, standard deviation, and upper and lower 95% confidence intervals for the timing, percentage contribution, and phase angle variables, respectively, during every condition of the exercise challenge for individuals with suspected BPD and HBP.

### 3.1. OEP Parameter Differences between Exercise Intensities for Breathing Pattern Disorder Group

#### 3.1.1. Rest Versus all Exercise Intensities

Within the group of individuals classified with BPD, the following breathing variables displayed significant differences between rest and exercise: RR, Vt, ${\dot{V}}_{E}$, tI, tE, tTot (Table 2), RcCT, AbCT, RCaAbCT, RcAbIndex (Table 3), RcAbPhase, RCpAbPhase, RCpSPhase, AbSPhase and inhale % deviation (Table 4).

#### 3.1.2. Rest Versus High Intensity

RcpRCaPhase. RCaSPhase, and exhale % deviation were found to be significantly different between rest and high exercise intensity only. RR, Vt, ${\dot{V}}_{E}$, tI, tE, tTot, RcAbPhase, and RCpAbPhase were significantly different between high exercise intensity and other exercise intensities (Tables 3 and 4).

#### 3.1.3. Rest Versus Recovery

Finally, RR, Vt, ${\dot{V}}_{E}$, tI, tE, tTot, RCpSPhase, and inhale % deviation were found to be significantly different between rest and recovery, with RR, Vt, ${\dot{V}}_{E}$, tI, tE, tTot, RCaCT, RcAbPhase, RCpAbPhase, AbSPhase, and exhale % deviation significantly different between recovery and some exercise intensities.

### 3.2. OEP Parameter Differences between BPD and HBP Groups

Tables 2–4 also display the comparison between individuals with and without BPD at rest, during exercise and recovery postexercise. At rest, only RCaCT. RcCT, AbCT, and RcAbIndex were found to be significantly different between the two groups (Table 3).

During high intensity exercise, the variables tI, tE, tTot, RCaCT, RcAbPhase, RCpRCaPhase, RCaSPhase, RCpSPhase, inhale % deviation and exhale % deviation displayed significant differences between healthy individuals and individuals with BPD.

**Figure 2:**
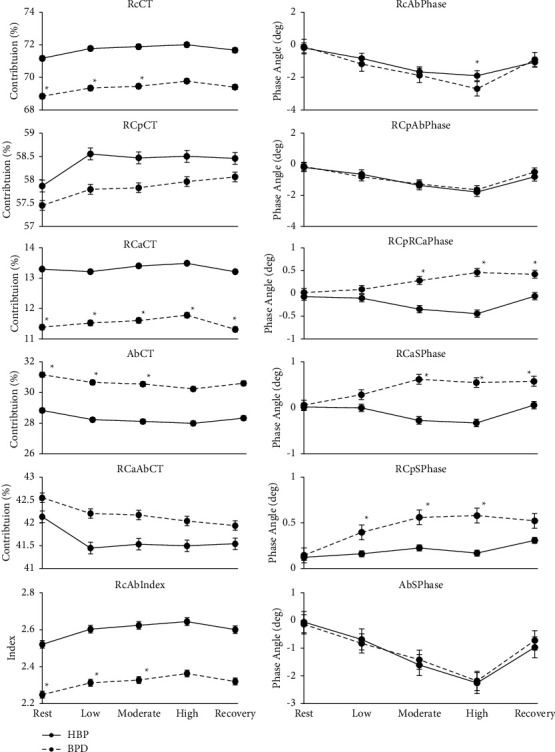
Illustration of contribution and phase angle variables exercise response for the healthy breathing pattern group (solid line) and the breathing pattern disorder group (dashed line). Significant difference between the groups with *p* < 0.05 is indicated with ^*∗*^.

During recovery, RCaCT, RCpRCaPhase, and RCaSPhase were found to be significantly different between the two groups, with all other variables demonstrating no significant difference.  Figure 2 displays how the regional contribution and phase angle variables changed across conditions for both groups.

## 4. Discussion

**Figure 3:**
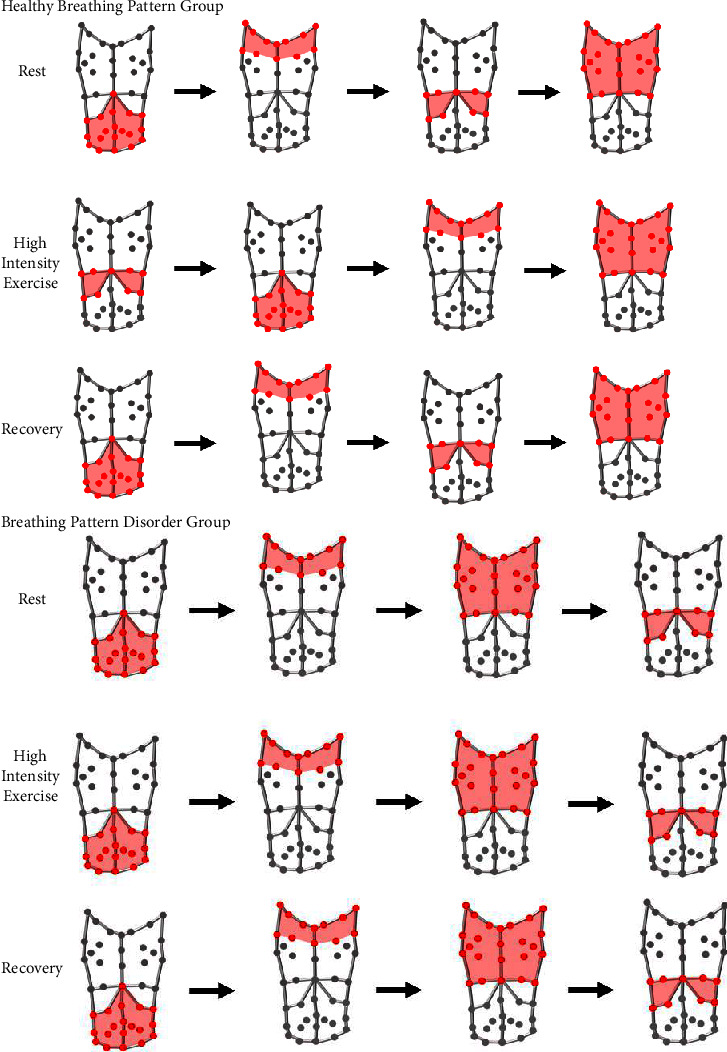
Illustration of the compartment movement order from left to right, for the HBP and BPD groups during rest, high intensity exercise, and recovery.

For the first time this study demonstrates that OEP-derived breathing variables, including rib cage and abdomen compartment movements and phase angles, discriminate between healthy and disordered breathing patterns. Healthy breathing during exercise appears to be initiated via movement in the lower rib cage (RCa) before the shoulders (Figure 3).

Our findings (Table 3) demonstrate that individuals classified into the BPD group used more abdominal and less ribcage contribution to total volume compared to the HBP group. Previous research has investigated these contribution variables using SLP at rest in asthmatic and chronic obstructive pulmonary disease (COPD) populations groups [20, 21]. Laveneziana and colleagues [20] found a reduction in SLP-derived ribcage contribution postbronchodilation in individuals with COPD.

Phase angles between the ribcage and abdomen (RcAbPhase) and pulmonary ribcage and abdomen (RCpAbPhase), and between the shoulder and the pulmonary ribcage and abdomen (RCpSPhase and AbSPhase respectively) demonstrated significant differences between rest and exercise within the group classified as BPD (Table 4). Similarly, the phase angle between the two ribcage sections (RCpRCaPhase) and between the abdominal ribcage and shoulder sections (RCaSPhase) displayed significant differences between rest and moderate and high exercise intensities, respectively (Table 4). For each phase angle, there was an increased in value as exercise intensity increased (Table 4), indicating that individuals classified with a BPD demonstrate a more asynchronous breathing pattern during exercise compared to rest. Overall, the group classified as BPD displayed larger phase angle values compared to HBP across each condition (Table 4). This indicates that the group classified as BPD had a significantly more asynchronous breathing pattern during exercise and recovery postexercise when compared to HBP.

More specifically, the phase angle between the ribcage and abdomen (RcAbPhase) was significantly different between the two groups during high intensity exercise only with the abdomen moving first followed by the ribcage in both groups. This phase angle is a measure of thoracoabdominal asynchrony which is a characteristic currently used to identify BPD, e.g., BPAT, and is widely used. Previously, SLP-derived RcAbPhase has been used to distinguish between children with BPD compared to a healthy group with the BPD group displaying lower phase angle values [22], which contrasts with the findings of this study. However, Barker and colleagues [22] reported the BPD group as having phase angles closer to zero compared to the control group but did not comment on the shape of the corresponding Konno–Mead loops. RIP-derived RcAbPhase has been used to identify BPD within individuals with severe asthma and, similar to this study, those with a BPD displayed larger phase angle values compared to controls [23]. Pereria and colleagues [24] also found that OEP and RIP-derived RCpAbPhase displayed more asynchrony in COPD patients compared to healthy individuals during rest and exercise. Similarly, SLP-derived RcAbPhase has been shown to be significantly larger in patients with COPD [20].

The phase angle between the two sections of the ribcage (RCpRCaPhase) demonstrated significant differences between the two groups during moderate and high intensity exercise and also recovery. OEP has previously been used to establish RCpRCaPhase exercise response in COPD patients [24, 25]. It is thought that changes in phase angles within COPD patients is dependent on the position of the diaphragm and the degree of activation of the inspiratory muscles [24, 26]. For RCpRCaPhase, the group classified as BPD displayed a breathing pattern with the RCp moving before the RCa; however, the HBP group displayed the opposite (Table 4). This indicates that the individuals classified as BPD displayed a thoracic dominant breathing pattern, which is a characteristic often used in assessments such as the BPAT to identify BPD. This OEP-derived phase angle offers an objective and quantitative measure of thoracic dominant breathing patterns.

During exercise, the phase angle between the pulmonary ribcage and shoulder sections (RCpSPhase) was significantly larger for individuals classified into the BPD group when compared to the HBP group and was significantly different between the two groups during moderate and high intensity exercise and also recovery. For RCpSPhase, the shoulders moved before the RCp in both groups across all conditions (Figure 3). For RCaSPhase, the shoulders moved first followed by the RCa across all conditions for the BPD group. However, for the HBP group, the RCa moved before the shoulders during exercise. This indicates the breath for the BPD group was not initiated by the RCa, yet this seems to be the initiating compartment in the HBP group during exercise.

These shoulder phase angles are novel variables, and therefore, comparison to other studies is not possible. They may offer additional information for assessing a apical or thoracic dominant breathing pattern which is a typical characteristic of breathing pattern disorder [5]. Thoracic dominant breathing patterns involve an increase in the vertical motion of the ribcage with minimal abdomen movement [6]. This motion can increase the activation of muscles of the upper ribcage such as the upper trapezius and may increase the elevation of the shoulders [27]. This study supports this mechanism with the group classified as BPD displaying shoulder movement prior to lower ribcage movement during a breath across every condition. This characteristic of a dysfunctional breathing pattern may cause postural issues and/or shoulder pain [27].

**Figure 4:**
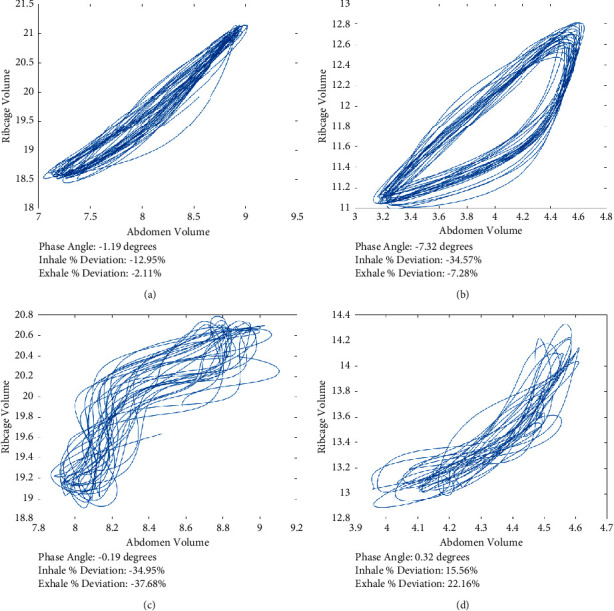
Examples of RcAbPhase Konno-Mead loops for (a) a healthy breathing pattern with a low phase angle and low inhale and exhale percentage deviation, (b) a breathing pattern disorder with a high phase angle and large deviations and (c, d) breathing pattern disorders with a low phase angle but large percentage deviations during high intensity exercise.

Although the group classified as BPD displayed significantly higher RcAbPhase values during high intensity exercise compared to the HBP group (Table 4), some of the participants within the BPD group displayed unexpectedly low RcAbPhase values similar to that of HBP. However, after visual inspection, the corresponding Konno–Mead loops did not display a typical HBP, i.e., close to a straight 45 degree diagonal line. The phase angle calculation is a measure of the synchrony between two compartments, but more specifically, it is a measure of the tightness of the Konno–Mead loop. For example, Figures 4(c), 4(d) display a Konno–Mead loop from the BPD group with a low phase angle; however, it does not follow a straight diagonal line. Within this plot (Figure 4(c)) specifically, it indicates that the abdomen is moving first out of synchrony with the ribcage during the inhale and the opposite is happening during the exhale resulting in the tight loop, and therefore, a low phase angle value is seen. This issue prompted the development of the novel variables inhale and exhale percentage deviation, which are measures of how much the inhale and exhale deviate from the straight 45-degree diagonal line within a Konno–Mead loop.

During high intensity exercise, the BPD group displayed significantly greater inhale (*p* ≤ 0.000) and exhale (*p* ≤ 0.011) percentage deviation values (Mean ± SD: inhale 19.7 ± 8.6%, exhale −17.6 ± 7.5%) when compared to the HBP group (Mean ± SD: inhale: 8.8 ± 6.4%, exhale: −10.9 ± 7.4%). The differences in Konno–Mead loop shape may be explained by different types of breathing patterns with BPD. Previous research has suggested that BPD may present differently between individuals such as thoracoabdominal asynchrony or thoracic dominant breathing [6]. The findings of this study provide a basis for further investigation into potential classifications within BPD with a more substantive data set.

The results thus far have shown that the contribution variables seem to be useful in distinguishing between these two population groups at rest and during the lower exercise intensities, but as exercise intensity increases, they become less important. In contrast, the phase angle variables seemed to be most useful during the higher exercise intensities and recovery postexercise but less useful during rest and low exercise intensity. This indicates that there is no one singular OEP-derived variable that could be used to distinguish between healthy and BPD at rest and during exercise. However, by using a combination of these variables, a complete picture can be obtained of an individual's breathing pattern. Generally, across rest and exercise, individuals classified as BPD seem to display lower RC contribution and higher AB contribution compared to HBP. They also demonstrate more asynchrony during exercise resulting in larger phase angle values with the shoulders, and RCp and Ab initiating the breath. However, depending on the type of breathing pattern disorder, an individual may display an unexpected low phase angle; therefore, the percentage deviation variables should be used to distinguish between a disordered low phase angle and a healthy low phase angle. These deviation variables can also give greater insight into where in the breath cycle, the asynchrony occurs. For example, Figure 4(b) displays an asynchronous BPD with greater deviation during the inhale (−34.57%) compared to the exhale (−7.28%).

Previous research has demonstrated that breathing pattern training within a BPD group improves symptoms and quality of life [14, 15]. Previously, OEP has been used as a real-time feedback system to acutely revert BPD towards a HPB by reducing RR, RcAbPhase, RCpAbPhase, and AbSPhase during exercise [28]. OEP-derived breathing variables including the novel deviation variables presented in this study may allow for more targeted and individualised breathing pattern training depending of the type of BPD.

A limitation of OEP is that in order to successfully track each marker, participants often have to extend their arms to the side. This is an unnatural position particularly during exercise and has the potential to alter the activation of inspiratory muscles and thus alters the breathing pattern. To minimise this, participants rested their arms on stands rather than actively holding them out.

There is no gold standard measure for the identification of BPD, which is currently done by exclusion of other conditions [13]. Participants were classified in this study into the BPD group if they had no diagnosis of underlying cardio-respiratory pathology, had a normal EVH challenge result yet reported respiratory symptoms such as wheezing or dyspnoea during exercise generally, and specifically during this exercise test. Nevertheless, the absence of an objective measure of BPD is a limitation. A future OEP-based diagnostic criteria for BPD may provide a more objective measure and so shorten time to diagnosis. However, it must be acknowledged that the present study is based on 26 BPD and 47 HBP participants. While this study provides insight into the differences between BPD and HBP, in order to establish clear cut-off criteria to objectively support the diagnosis of BPD, a more substantive data set would be required, particularly given the different clusters of disordered breathing pattern highlighted here in Figure 4 and reported elsewhere.

The mean age of the females in the BPD group was higher than the mean age of the HBP group although the characteristics of the males in each group were similar. However, there is no clear evidence that older females in the general UK population may be more susceptible to experience exercise respiratory symptoms and BPD, or that age in females is likely to affect the measures of breathing pattern considered here. We recommend this may be an area for further study.

## 5. Conclusion

This study demonstrates that OEP can be used to characterise breathing pattern disorder and distinguish between individuals with and without a BPD at rest and during exercise. At rest, the contribution variables (RcCT, RCaCT, AbCT, and RcAbIndex) were significantly different between HBP and BPD, with the BPD group displaying less ribcage and more abdominal contribution. During exercise, the BPD displayed significantly more asynchrony between various thoracic compartments (RcAbPhase, RCpRCaPhase, RCaSPhase, and RCpSPhase) such that thoracoabdominal asynchrony and thoracic dominant breathing patterns were identified in the BPD group. However, it is clear that there is no one singular OEP-derived breathing variable that could be used to distinguish between healthy and BPD, but by using a combination of these variables, a greater insight can be obtained and used to distinguish between these population groups. This includes the newly developed inhale and exhale percentage deviation variables that successfully distinguished the groups during high intensity exercise. Our findings suggest that OEP is capable of identifying differences between conditions within a population but also between populations, providing a basis for further investigation into OEP as a potential diagnostic and training tool.

## Figures and Tables

**Table 1:** Participant information (mean ± SD).

	*N*	Age (yrs)	Height (m)	Weight (kg)	Activity level (hrs/week)
Healthy breathing pattern group *n* = 47					
Male	28	34.4 ± 12.1	1.8 ± 0.1	77.8 ± 11.5	5.6 ± 2.7
Female	19	28.4 ± 9.3	1.7 ± 0.1	61.3 ± 5.5	5.3 ± 2.6

Breathing pattern disorder group *n* = 26					
Male	12	34.8 ± 9.6	1.8 ± 0.1	75.7 ± 7.4	5.2 ± 2.5
Female	14	40.9 ± 11.5	1.6 ± 0.1	58.8 ± 6.4	7.1 ± 4.2

**Table 2:** Mean, standard deviation (SD), and 95% confidence intervals for the timing breathing variables across each condition for individuals with a healthy breathing pattern and breathing pattern disorder. Significant differences between conditions are represented by ^*∗*^, +, #, †, and ^ as a mean significant (*p* < 0.05) difference from rest, low, moderate, high intensity exercise, and recovery, respectively. Significant difference between the two groups is indicated by ^*∗∗*^.

Variable	Condition	Healthy breathing Pattern				Breathing Pattern Disorder				Comparison between groups
		Mean	SD	95% CI		Mean	SD	95% CI		
				Lower	Upper			Lower	Upper	*p* value
RR (brpm)	Rest	15.36^+#†^^	3.51	14.36	16.36	15.62^+#†^^	3.94	14.11	17.14	0.951
	Low	21.82^*∗*^^#†^	4.80	20.46	23.20	22.63^*∗*^^#†^	4.36	20.96	24.31	0.481
	Moderate	24.84^*∗*^^+†^	5.01	23.40	26.27	26.92^*∗*^^+†^	4.96	25.01	28.82	0.093
	High	33.15^*∗*^^+#^^	6.59	31.26	35.03	36.93^*∗*^^+#^^	8.37	33.71	40.15	0.127
	Recovery	23.37^*∗*^^†^	4.69	22.03	24.71	23.29^*∗*^^†^	5.70	21.10	25.48	0.997

Vt(litres)	Rest	0.75^+#†^^	0.33	0.65	0.84	0.70^+#†^^	0.33	0.57	0.83	0.827
	Low	1.36^*∗*^^#†^^	0.30	1.28	1.45	1.72^*∗*^^#†^	0.63	1.47	1.96	0.011^*∗∗*^
	Moderate	2.09^*∗*^^+†^	0.67	1.90	2.29	2.18^*∗*^^+†^	0.76	1.89	2.47	0.637
	High	2.64^*∗*^^+#^^	0.86	2.40	2.89	2.60^*∗*^^+#^^	0.95	2.24	2.97	0.985
	Recovery	1.83^*∗*^^+†^	0.53	1.68	1.98	1.95^*∗*^^†^	0.71	1.68	2.23	0.722

${\dot{V}}_{E}\left(lit/\;\min\;\right)$	Rest	11.19 ^+#†^^	4.42	9.93	12.46	10.20^+#†^^	4.78	8.37	12.04	0.620
	Low	28.75^*∗*^^#†^^	6.66	26.85	30.66	37.94^*∗*^^#†^	14.07	32.54	43.35	0.004^*∗∗*^
	Moderate	49.96^*∗*^^+†^	14.98	45.68	54.25	55.76^*∗*^^+†^	17.11	49.18	62.34	0.137
	High	85.35^*∗*^^+#^^	32.27	76.13	94.58	93.70^*∗*^^+#^^	38.40	78.94	108.46	0.618
	Recovery	41.38^*∗*^^+†^	14.69	37.18	45.58	46.23^*∗*^^†^	22.55	37.56	54.60	0.589

tI(s)	Rest	1.85^+#†^^	0.47	1.72	1.99	1.86^+#†^^	0.33	1.73	1.98	1.000
	Low	1.37^*∗*^^#†^	0.33	1.28	1.46	1.24^*∗*^^#†^	0.19	1.17	1.31	0.031^*∗∗*^
	Moderate	1.22^*∗*^^+†^	0.24	1.15	1.29	1.08^*∗*^^+†^	0.16	1.02	1.14	0.004^*∗∗*^
	High	0.94^*∗*^^+#^^	0.24	0.87	1.01	1.31^*∗*^^+#^^	0.37	1.20	1.41	0.010^*∗∗*^
	Recovery	0.80^*∗*^^†^	0.15	0.75	0.86	1.24^*∗*^^†^	0.28	1.13	1.35	0.716

tE(s)	Rest	2.37^+#†^^	0.66	2.18	2.56	2.38^+#†^^	0.60	2.15	2.61	0.998
	Low	1.54^*∗*^^#†^	0.39	1.43	1.65	1.35^*∗*^^†^	0.25	1.25	1.45	0.014^*∗∗*^
	Moderate	1.32^*∗*^^+†^	0.28	1.24	1.40	1.12^*∗*^^†^	0.19	1.05	1.20	0.001^*∗∗*^
	High	0.99^*∗*^^+#^^	0.27	0.91	1.07	0.84^*∗*^^+#^^	0.17	0.77	0.90	0.011^*∗∗*^
	Recovery	1.44^*∗*^^†^	0.42	1.32	1.56	1.33^*∗*^^†^	0.30	1.21	1.45	0.481

tTot(s)	Rest	4.23^+#†^^	1.08	3.92	4.54	4.31^+#†^^	0.93	3.95	4.67	0.946
	Low	2.90^*∗*^^#†^	0.70	2.70	3.10	2.57^*∗*^^#†^	0.43	2.40	2.73	0.014^*∗∗*^
	Moderate	2.54^*∗*^^+†^	0.53	2.39	2.69	2.20^*∗*^^+†^	0.35	2.06	2.33	0.001^*∗*^
	High	1.94^*∗*^^+#^^	0.52	1.79	2.09	1.65^*∗*^^+#^^	0.31	1.53	1.77	0.012^*∗∗*^
	Recovery	2.75^*∗*^^†^	0.77	2.52	2.97	2.57^*∗*^^†^	0.58	2.35	2.79	0.591

**Table 3:** Mean, standard deviation (SD), and 95% confidence intervals for the percentage contribution breathing variables across each condition for individuals with a healthy breathing pattern and breathing pattern disorder. Significant differences between conditions are represented by ^*∗*^, +, #, †, and ^ as a mean significant (*p* < 0.05) difference from rest, low, moderate, high intensity exercise, and recovery, respectively. Significant difference between the two groups is indicated by ^*∗∗*^.

Variable	Condition	Healthy breathing pattern				Breathing pattern disorder				Comparis between groupson
		Mean	SD	95% CI		Mean	SD	95% CI		
				Lower	Upper			Lower	Upper	*p* value
RCpCT (%)	Rest	57.87^+#†^^	4.56	56.56	59.17	57.45	4.70	55.65	59.26	0.925
	Low	58.55^*∗*^	4.56	57.25	59.85	57.79	5.12	55.83	59.76	0.517
	Moderate	58.47^*∗*^	4.93	57.06	59.88	57.83	5.18	55.84	59.82	0.604
	High	58.50^*∗*^	4.83	57.12	59.88	57.96	5.22	55.95	59.97	0.891
	Recovery	58.46^*∗*^	4.89	57.06	59.86	58.06	5.29	56.03	60.09	0.942

RCaCT (%)	Rest	13.30	2.42	12.60	13.99	11.38	1.89	10.66	12.11	0.002^*∗∗*^
	Low	13.21^†^	2.56	12.56	13.86	11.52	2.08	10.72	12.32	0.003^*∗∗*^
	Moderate	13.40	2.51	12.68	14.12	11.60	2.16	10.78	12.43	0.003^*∗∗*^
	High	13.49^+^	2.38	12.81	14.17	11.78^^^	2.15	10.95	12.61	0.007^*∗∗*^
	Recovery	13.21	2.39	12.52	13.89	11.31^†^	2.17	10.48	12.15	0.003^*∗∗*^

RcCT (%)	Rest	71.17^+#†^^	3.54	70.16	72.18	68.84^+#†^	3.44	67.52	70.16	0.022^*∗∗*^
	Low	71.77^*∗*^	3.64	70.73	72.81	69.34^*∗*^	3.80	67.88	70.80	0.009^*∗∗*^
	Moderate	71.88^*∗*^	3.81	70.80	72.97	69.45^*∗*^	3.89	67.96	70.95	0.012^*∗∗*^
	High	72.01^*∗*^^^^	3.91	70.89	73.12	69.77^*∗*^	3.89	68.27	71.26	0.053
	Recovery	71.67^*∗*^^†^	3.97	70.53	72.80	69.40	3.86	67.91	70.88	0.054

AbCT (%)	Rest	28.83^+#†^^	3.52	27.82	29.84	31.16^+#†^	3.44	29.84	32.48	0.022^*∗∗*^
	Low	28.23^*∗*^	3.64	27.19	29.27	30.66^*∗*^	3.80	29.20	32.12	0.009^*∗∗*^
	Moderate	28.12^*∗*^	3.81	27.03	29.20	30.55^*∗*^	3.89	29.05	32.04	0.012^*∗∗*^
	High	27.99^*∗*^^^^	3.91	26.88	29.11	30.23^*∗*^	3.89	28.74	31.72	0.053
	Recovery	28.33^*∗*^^†^	3.97	27.20	29.47	30.60	3.86	29.12	32.09	0.054

RCaAbCT (%)	Rest	42.13^+#†^^	4.56	40.83	43.44	42.55^+#†^	4.70	40.74	44.36	0.925
	Low	41.45^*∗*^	4.56	40.15	42.75	42.21^*∗*^	5.12	40.24	44.17	0.517
	Moderate	41.53^*∗*^	4.93	40.12	42.94	42.17^*∗*^	5.18	40.18	44.17	0.604
	High	41.50^*∗*^	4.83	40.12	42.88	42.04^*∗*^	5.22	40.03	44.05	0.891
	Recovery	41.54^*∗*^	4.89	40.14	42.94	41.94	5.29	39.91	43.97	0.942

RcAbIndex	Rest	2.52^+#†^^	0.44	2.39	2.65	2.25^+#†^	0.37	2.11	2.39	0.025^*∗∗*^
	Low	2.60^*∗*^	0.49	2.46	2.74	2.32^*∗*^	0.43	2.15	2.48	0.013^*∗∗*^
	Moderate	2.62^*∗*^	0.51	2.48	2.77	2.33^*∗*^	0.44	2.16	2.50	0.015^*∗∗*^
	High	2.64^*∗*^^^^	0.54	2.49	2.80	2.36^*∗*^	0.45	2.19	2.54	0.060
	Recovery	2.60^*∗*^^†^	0.53	2.45	2.75	2.32^*∗*^	0.43	2.15	2.48	0.057

**Table 4:** Mean, standard deviation (SD), and 95% confidence intervals for the phase angle breathing variables across each condition for individuals with a healthy breathing pattern and breathing pattern disorder. Significant differences between conditions are represented by ^*∗*^, +, #, †, and ^ as a mean significant (*p* < 0.05) difference from rest, low, moderate, high intensity exercise, and recovery, respectively. Significant difference between the two groups is indicated by ^*∗∗*^.

Variable	Condition	Healthy breathing pattern				Breathing pattern disorder				Comparison between groups
		Mean	SD	95% CI		Mean	SD	95% CI		
				Lower	Upper			Lower	Upper	*p* value
RcAbPhase (deg)	Rest	−0.20^+#†^^	0.39	−0.31	−0.09	−0.12^+#†^	0.53	−0.32	0.08	0.749
	Low	−0.84^*∗*^^#†^	0.76	−1.06	−0.62	−1.19^*∗*^^†^	0.92	−1.54	−0.83	0.086
	Moderate	−1.66^*∗*^^+†^^	0.99	−1.95	−1.38	−1.88^*∗*^^†^^	1.01	−2.27	−1.49	0.377
	High	−1.90^*∗*^^+#^^	1.06	−2.20	−1.60	−2.70^*∗*^^+#^^	1.71	−3.36	−2.04	0.039^*∗∗*^
	Recovery	−1.07^*∗*^^#†^	0.90	−1.33	−0.81	−0.91^#†^	1.12	−1.34	−0.48	0.771

RCpAbPhase (deg)	Rest	−0.20^+#†^^	0.42	−0.32	−0.08	−0.14^+#†^	0.44	−0.31	0.03	0.830
	Low	−0.65^*∗*^^#†^	0.70	−0.84	−0.44	−0.81^*∗*^^†^	0.76	−1.10	−0.51	0.359
	Moderate	−1.37^*∗*^^+†^^	0.87	−1.61	−1.12	−1.27^*∗*^^^^	0.99	−1.65	−0.89	0.667
	High	−1.79^*∗*^^+#^^	1.28	−2.16	−1.43	−1.64^*∗*^^+^^	1.43	−2.19	−1.20	0.899
	Recovery	−0.81^*∗*^^#†^	0.94	−1.08	−0.54	−0.50^#†^	0.95	−0.87	−0.14	0.361

RCpRCaPhase (deg)	Rest	−0.07^†^	0.28	−0.15	0.01	0.02^†^	0.24	−0.08	0.11	0.332
	Low	−0.11	0.59	−0.27	0.06	0.09	0.61	−0.15	0.32	0.181
	Moderate	−0.35	0.82	−0.58	−0.11	0.28	0.80	−0.03	0.58	0.002^*∗∗*^
	High	−0.45^*∗*^^^^	1.08	−0.75	−0.14	0.46^*∗*^	0.82	0.14	0.77	0.001^*∗∗*^
	Recovery	−0.06^†^	0.80	−0.29	0.17	0.42	0.72	0.14	0.70	0.025^*∗∗*^

RCaSPhase (deg)	Rest	0.02	0.27	−0.05	0.10	0.06^#^	0.29	−0.05	0.17	0.813
	Low	0.00	0.57	−0.16	0.16	0.29	0.81	−0.02	0.60	0.082
	Moderate	−0.27	0.91	−0.53	−0.01	0.62^*∗*^	0.80	0.31	0.93	<0.001^*∗∗*^
	High	−0.33^^^	1.26	−0.69	0.03	0.55	0.93	0.19	0.91	0.005^*∗∗*^
	Recovery	0.06^†^	0.93	−0.21	0.33	0.58	0.91	0.23	0.93	0.049^*∗∗*^

RCpSPhase (deg)	Rest	0.12	0.19	0.07	0.18	0.14^+#†^^	0.22	0.06	0.23	0.887
	Low	0.16	0.36	0.06	0.26	0.40^*∗*^	0.42	0.23	0.56	0.014^*∗∗*^
	Moderate	0.40	0.43	0.09	0.36	0.56^*∗*^	0.48	0.38	0.74	0.006^*∗∗*^
	High	0.17	0.67	−0.02	0.36	0.58^*∗*^	0.54	0.37	0.79	0.022^*∗∗*^
	Recovery	0.31	0.45	0.18	0.44	0.52^*∗*^	0.64	0.27	0.77	0.200

AbSPhase (deg)	Rest	−0.06^+#†^^	0.43	−0.18	0.07	−0.14^#†^	0.61	−0.37	0.10	0.784
	Low	−0.68^*∗*^^#†^	0.89	−0.94	−0.43	−0.83^†^	1.14	−1.27	−0.39	0.552
	Moderate	−1.61^*∗*^^+†^^	1.25	−1.96	−1.25	−1.42^*∗*^	1.05	−1.82	−1.01	0.511
	High	−2.26^*∗*^^+#^^	1.70	−2.74	−1.77	−2.18^*∗*^^+^^	1.79	−2.87	−1.49	0.983
	Recovery	−0.97^*∗*^^#†^	1.20	−1.31	−0.63	−0.72^†^	1.32	−1.23	−0.21	0.671

Inhale % deviation	Rest	−0.11^+#†^^	11.07	−3.03	2.81	1.33^+#†^^	14.82	−4.37	7.03	0.017
	Low	4.64^*∗*^^#^^	10.27	1.718	7.55	10.39^*∗*^	11.17	6.51	14.39	0.002^*∗∗*^
	Moderate	9.60^*∗*^+	10.36	6.68	12.52	13.66^*∗*^	11.49	8.25	18.50	0.004^*∗∗*^
	High	8.80^*∗*^	8.40	5.95	11.79	19.72^*∗*^	8.60	15.94	22.25	0.000^*∗∗*^
	Recovery	13.06^*∗*^^	8.64	10.14	15.97	12.06^*∗*^	12.50	7.26	16.87	0.093

Exhale % deviation	Rest	−5.48^+#†^	9.50	−8.40	−2.55	−3.34 †	14.30	−8.94	2.05	0.999
	Low	−10.64^*∗*^^^^	10.09	−13.57	−7.72	−8.38	11.34	−4.77	−5.03	0.839
	Moderate	−13.47^*∗*^^^^	10.66	−16.39	−10.54	−10.18^^^	11.34	−5.99	−6.52	0.665
	High	−10.90^*∗*^^^^	7.41	−13.77	−7.92	−17.60^*∗*^^^^	7.50	−20.42	−14.21	0.011^*∗∗*^
	Recovery	−4.66^+#†^	11.54	−7.58	−1.73	−2.00^#†^	16.20	−8.20	4.25	0.937

## Data Availability

The data used to support the findings of the study can be obtained from the corresponding author upon request.
